# *Toxoplasma gondii* and Rabies—The Parasite, the Virus, or Both?

**DOI:** 10.3390/microorganisms13010109

**Published:** 2025-01-08

**Authors:** Ragan Wilson, Shannon Caseltine, Edith Will, Jeremiah Saliki, Ruth C. Scimeca

**Affiliations:** 1 Department of Veterinary Pathobiology, Oklahoma State University, Stillwater, OK 74078, USA; 2 Oklahoma Animal Diseases Diagnostic Laboratory, Oklahoma State University, Stillwater, OK 74078, USA

**Keywords:** protozoan, virus, neurological signs, one health

## Abstract

*Toxoplasma gondii* is an intracellular protozoan parasite that infects a wide range of vertebrates, including humans. Although cats are the only definitive host, any warm-blooded animal can act as a paratenic host. Throughout the years, this apicomplexan parasite has been studied due to its wide prevalence, zoonotic potential, and host behavioral alterations. Known for its neurological alterations, the rabies virus is one of the most recognized types of zoonosis that, although preventable, still causes deaths in humans and animals worldwide. Due to the overlapping clinical signs of these two pathogens, the objective of this study was to evaluate the prevalence of *T. gondii* DNA in cerebellum tissue collected for rabies testing; cerebellum tissue from diverse animals is often submitted for this purpose. Between May 2022 and April 2024, we tested 903 cerebellum tissue samples from 22 animal species submitted for rabies testing to the Oklahoma Animal Diagnostic Disease Laboratory. Overall, *T. gondii* prevalence was 3.96%, with 1.8% found in cats (*Felis catus*), 1.7% in dogs (*Canis familiaris*), 0.3% in skunks (*Mephitis mephitis*), and 0.2% in infected cattle (*Bos taurus*). Analysis among *T. gondii*-positive hosts revealed a statistically significant difference in dogs when comparing neutered vs. intact males, with 7.94% (5/63) *T. gondii*-positive neutered males and 1.61% (3/186) *T. gondii*-positive intact males (*p* = 0.02). All the *T. gondii*-positive samples were negative for rabies. Anamnesis in some of the *T. gondii*-positive samples included ataxia, aggression, muscle rigidity, lethargy, and seizures, with the latter also described in dogs and aggression in the positive bovine sample. The clinical signs described in the *T. gondii*-infected hosts can be mistaken for rabies infection; therefore, it is important to consider *T. gondii* as a differential diagnosis in suspected rabies cases and test for this parasite when negative rabies results are obtained.

## 1. Introduction

*Toxoplasma gondii (T. gondii)* is an intracellular protozoan parasite that infects mammals and birds and utilizes felids as definitive hosts. It is the causative agent of toxoplasmosis, a worldwide zoonosis, and one of the most important foodborne parasites in the USA with a high economic burden [1,2]. Studies on the seroprevalence of *T. gondii*—IgG and IgM antibodies are the most common way to evaluate the status of infection or exposure to this parasite. In the USA, its prevalence in humans has been reported by the National Health and Nutrition Examination Survey (NHANES). In the past decade, the highest prevalence reported was 12.8% between 2011 and 2012, reflecting a decrease compared to previous years [3]. Domestic animals are also monitored for *T. gondii* seroprevalence due to their close relationship to humans, either as pets or for food consumption, and to monitor clinical disease. Dogs and cats have been reported to have different seroprevalence levels among populations, with dogs varying between 21% and 42.8% according to location and lifestyle [4] and cats between 28% and 43% [5].

Animals and humans become infected by ingesting water or food contaminated with oocysts excreted by cats or ingesting bradyzoites in undercooked meat, raw meat, milk, or transplacentally. After infection in the intermediate host, *T. gondii* freely passes through the intestinal or placental epithelium and enters macrophages or dendritic cells, distributing to different organs [6,7]. The parasite then either rapidly replicates (tachyzoites) or forms cysts in the host’s tissues (bradyzoites), with skeletal muscle and the brain being two of the most common organs hosting the slow-replicating form.

Although acute toxoplasmosis is uncommon, several comorbidities, the immune status of the host, and the parasite genotype, among others, can contribute to the severity of the disease. Due to the wide range of hosts that can be infected by *T. gondii*, its biology, zoonotic implications, and host behavioral alterations have been broadly studied [8,9,10,11,12]. In humans, toxoplasmosis has been linked to congenital and neuropsychiatric disorders [13,14], cognitive impairment, and increment of dopamine metabolism in neural cells [10,15]. The status of seropositivity has also been associated with higher aggression and impulsivity [16]. In animals, various reports of neurological signs exist, as well as behavioral disorders such as anxiety and aggression [17,18,19]. In addition to affecting the health of humans and animals, it has an economic impact on farm animals due to the cost of biosecure management systems and clinical disease [18].

The purpose of this study was to analyze cerebellum samples previously tested for rabies virus for comorbidity with *T. gondii* in domestic and wild animals from Oklahoma. Additionally, we evaluated if *T. gondii* DNA detected in the cerebellum of various hosts can be associated with clinical signs such as those caused by the rabies virus. We selected these pathogens due to their zoonotic concern and capacity to cause neurological signs. The analysis presented in this study utilized samples collected from the state of Oklahoma, USA, during the years 2022 to 2024. We assessed 22 different hosts, summarizing the prevalence of both pathogens during that timeframe and describing the most common clinical signs observed in *T. gondii*-positive animals.

## 2. Materials and Methods

### 2.1. Sample Collection

Cerebellum tissue samples collected between May 2022 and April 2024 from 903 animals representing 22 different hosts (Table 1) were used in this study. After testing for the rabies virus at the Oklahoma Animal Disease Diagnostic, Stillwater, OK, USA, a small portion of the original sample (15–25 g) was placed in 70% ethanol and kept frozen at −80 °C until further processing. The clinical signs included were obtained from the rabies submittal form records provided by owners or veterinarians.

### 2.2. DNA Extraction

DNA was isolated from 3 to 5 mg of cerebellum tissue using the Quick-DNA miniprep plus kit (Zymo Research, Irvine, CA, USA), according to the manufacturer’s instructions. The concentration and quality of the extracted DNA were evaluated using Nanodrop (ND 8000, Thermo Fisher Scientific, Whatman, MA, USA).

### 2.3. Toxoplasma gondii PCR Amplification

We utilized a previously described nested PCR, which amplified a fragment of the *B1* gene for *T. gondii* DNA [20,21], using a total of 20 to 100 ng of genomic DNA. Positive control DNA was obtained from *T. gondii* RH strain maintained in cell culture. Nuclease-free water was used as a negative control.

### 2.4. Primary PCR Amplification

The primers Tg1 forward (5′ TGT TCT GTC CTA TCG CAA CG) and Tg2 reverse (5′ ACG GAT GCA GTT CCT TTC TG), specific for the *B1* gene were used for this PCR. Amplification was carried out using the GoTaq colorless master mix (Promega, Madison, WI, USA), 0.5 μL of bovine serum albumin (BSA, Thermo Fisher Scientific, Whatman, MA, USA), 10 pmol of forward and reverse primers, and 2 μL of DNA template, using nuclease-free water to adjust the total reaction volume to 25 μL. PCR was performed in a Bio-Rad T100 thermocycler (Hercules, CA, USA) with initial denaturation at 94 °C for 3 min, followed by 35 cycles of denaturation at 94 °C for 30 s, annealing at 60 °C for 45 s, extension at 72 °C for 45 s, and a final extension at 72 °C for 7 min. The product of the primary PCR consisted of a 580 bp fragment.

### 2.5. Secondary PCR Amplification

One μL from the primary PCR was used for the nested PCR with the following internal primer pair: Tg3 (5′ TCT TCC CAG ACG TGG ATT TC) and Tg4 (5′ CTC GAC AAT ACG CTG CTT GA). The nested PCR amplified a 531 bp DNA fragment. Apart from the primer pairs and DNA template, the reaction mixtures and cycling conditions remained the same for both rounds of PCR. The PCR products were visualized in 1.5% agarose using a GelRed Nucleic Acid Stain (Biotium, Fremont, CA, USA).

### 2.6. Neospora caninum PCR

Canine samples were additionally tested for *N. caninum*, a protozoan parasite that can cause similar clinical signs to *T. gondii* in canids. We utilized a real-time PCR using the TaqMan system previously described by Ghalmi [22] and a positive control obtained from DNA isolated from cell culture. A total of 430 samples were analyzed, with no amplification detected in any of the samples.

### 2.7. Sequencing of PCR Products

To confirm the presence of *T. gondii*, the PCR products from positive samples were purified using the GeneJet PCR purification kit (Thermo Fisher Scientific, Waltham, MA, USA) according to the manufacturer’s instructions and submitted for Sanger sequencing of the *B1* gene (Eurofins Genomics, Louisville, KY, USA). The results were analyzed with Geneious Prime software, version 2024.0.2, compared with available sequences at GenBank, and deposited under the accession number PQ723105.

### 2.8. Data Analysis

The prevalence of *T. gondii* infection was calculated according to Bush et al., 1997 [23]. The proportion of infected hosts according to sex was compared using Fisher’s exact tests. The results were considered statistically significant when *p* ≤ 0.05 [24]. Comparisons were performed using the statistical functions in Sigma Plot 12.5 (Sysat Software 2013). Maps were generated with Tableau 2024.1 (Seattle, WA, USA).

## 3. Results

**Figure 1:**
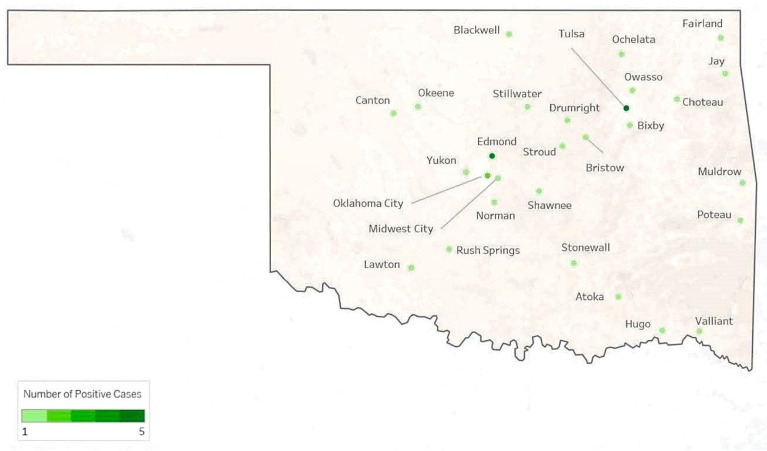
*Toxoplasma gondii* positive cases displayed by location in the state of Oklahoma.

**Figure 2:**
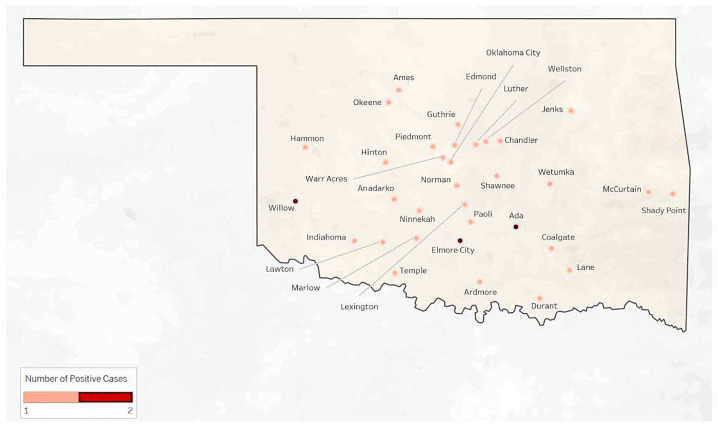
Rabies positive cases displayed by location in the state of Oklahoma.

A total of 903 samples from 22 different hosts were tested for *T. gondii* by PCR, utilizing DNA isolated from cerebellum tissue. The samples that tested positive for *T. gondii* originated from 28 different cities in the state of Oklahoma (OK, Figure 1), while samples that were positive for rabies originated from 33 locations (OK, Figure 2). None of the rabies-positive animals were PCR-positive for *T. gondii* (Table 1); however, the clinical signs described for *T. gondii*-positive animals were similar to those observed in rabies infection, with cats displaying a different subset of clinical signs or physical findings when compared to the other hosts (Table 2).

### 3.1. Toxoplasma gondii

**Figure 3:**
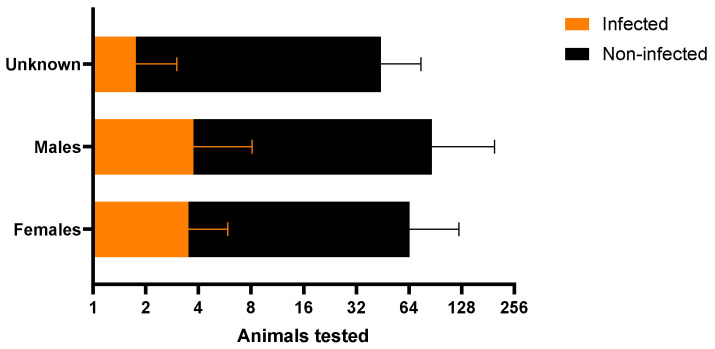
Infected and non-infected hosts that tested positive for *T. gondii.* according to sex.

The overall prevalence across hosts was 3.98% (36/903), with only bovine, dogs, cats, and skunks testing positive by PCR (Table 1). A comparison between females and males did not reveal significant differences (*p* = 0.56; Figure 3).

#### 3.1.1. Dogs

Of the 430 dogs tested, 139 were female, and 249 were male (42 did not have information on sex). There was no significant difference in the prevalence of. *T. gondii* between sexes, with 3.21% (8/249) of males testing positive and 3.57% (5/139) females testing positive (*p* = 0.99). When comparing the prevalence of *T. gondii* between spayed vs. intact females, no significance was found, with 2.78% (1/36) of positive spayed females testing positive and 3.88% (4/103) of positive intact females (*p* = 0.99). However, a statistically significant difference was found among neutered vs. intact males, with 7.94% (5/63) of *T. gondii*-positive neutered males and 1.61% (3/186) of *T. gondii*-positive intact males (*p* = 0.02).

#### 3.1.2. Cats

A total of 253 cats were tested, comprising 89 females and 87 males (77 did not have information on sex). There was no significant difference in the prevalence of. *T. gondii* between sexes, with 8.05% (7/87) of males testing positive and 6.74% (6/89) females testing positive (*p* = 0.78). When comparing the prevalence of *T. gondii* between spayed vs. intact females, no significance was found, with 15.79% (3/19) of positive spayed females and 4.29% (3/70) of positive intact females (*p* = 0.10). No statistical significance was found either among neutered vs. intact males, with non-neutered males being *T. gondii*-positive and 10.29% (7/68) of *T. gondii*-positive intact males (*p* = 0.33).

#### 3.1.3. Bovine

There was no significant difference in the prevalence of rabies between sexes, with 2.63% (0/10) of males testing positive and 7.89% (2/28) of females testing positive (*p* = 0.99).

### 3.2. Rabies

**Figure 4:**
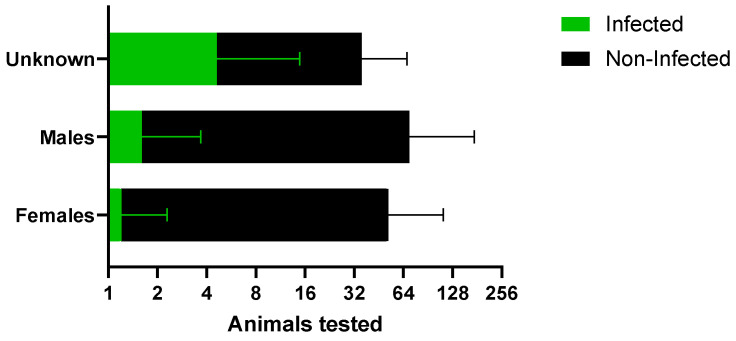
Infected and non-infected hosts that tested positive for rabies according to sex.

The overall prevalence was 3.6% (37/903) (Table 1), and no statistical difference was observed among females at 1.1% (6/511) and males at 2.5% (9/348) (*p* = 0.18); however, most positive rabies cases were skunks without information on sex. When comparing only females and males in hosts that tested positive for rabies, no statistical difference was found either (Figure 4). Reported clinical signs in rabies-positive animals are included in Table 3.

#### 3.2.1. Dogs

There was no significant difference in the prevalence of rabies between sexes, with 1.97% (5/249) of males testing positive and no females testing positive.

#### 3.2.2. Cats

There was no significant difference in the prevalence of rabies between sexes, with 2.3% (2/87) of males testing positive and 1.12% (1/89) of females testing positive (*p* = 0.61). When comparing the prevalence of rabies between spayed or neutered vs. intact cats, no significance was found with non-spayed females or neutered males found to be rabies-positive.

#### 3.2.3. Bovine

Thirty-nine bovines were tested, comprising 28 females and 10 males (1 did not have information on sex). There was no significant difference in the prevalence of. *T. gondii* between sexes, with no males testing positive and 7.14% (2/28) of females testing positive (*p* = 0.22).

#### 3.2.4. Skunks and Deer

A total of 58 skunks and 4 deer were tested. Aside from the prevalence analysis carried out, no statistical analysis was performed due to the lack of information regarding age or sex (Table 1).

## 4. Discussion

We report here the prevalence of *T. gondii* in 22 different hosts in the state of Oklahoma, utilizing samples collected over a period of two years. The detection of parasitic DNA in blood can only be used as a diagnostic method in a short period of time; therefore, most of the *T. gondii* prevalence reports are based on antibody detection [5]. In this study, we utilized PCR to detect DNA from cerebellum tissue submitted for rabies testing. Both pathogens tested have a zoonotic aspect and a tropism for the central nervous system, causing an overlap in clinical signs. The overall prevalence of *T. gondii* at 3.9% indicates the presence of the parasite in the central nervous system (CNS), which can produce significant alterations in the host behavior.

The clinical signs reported in some hosts indicate that infection with *T. gondii* can be mistaken for rabies infection and should be emphasized as a differential. Aggression, which is one of the main reasons for submission for rabies virus testing, was described in the domestic hosts detected as positive for *T. gondii* (Table 2 and Table 3). Dogs and cats were reported as having seizures. Interestingly, *T. gondii* has been associated with epilepsy and linked to the presence of cysts in the brain, followed by scar tissue formation [25]. Other clinical signs reported here, such as diarrhea and fever, were described in cats only. Although rare, clinical signs in cats infected with *T. gondii* are caused by inflammation and tissue necrosis due to replication and dissemination of tachyzoites. More commonly, clinical manifestations are due to immunosuppression and reactivation of latent infection [26]. After infection, lymphocytes produce cytokines that help to control the infection but, at the same time, lead to parasite latency with potential neurological consequences. In this study, cats had the highest prevalence among hosts (Table 1), highly likely to be related to the life cycle of the parasite. Dogs were the second host with a higher number of positive cases; they are unlikely to have clinical toxoplasmosis, and other pathogens such as distemper virus, *Sarcocystis neurona*, and *Neospora caninum* are oftentimes involved in clinical manifestations [27,28]. None of the dogs were detected as being *Neospora caninum*-infected by real-time PCR. Comparison between neutered vs. intact male dogs revealed a statistically significant difference, with 7.94% (5/63) identified as *T. gondii*-positive neutered males and 1.61% (3/186) as *T. gondii*-positive intact males (*p* = 0.02). Although we cannot explain this difference, hormonal changes could occur in infected dogs; previous studies have demonstrated alterations in the endocrine system of rodents infected with *T. gondii*, demonstrating that castrated male rodents have higher levels of testosterone and a reduced aversion to the odor of cat urine when compared to non-infected rodents [29,30]. Previous studies comparing dog populations have described pet dogs having higher *T. gondii* seroprevalence when compared to shelter dogs [4], a result which could be explained by the closer contact among dogs and cats in a household and could also represent a greater number of neutered pet dogs in proximity to cats.

Among the production animals tested, only cattle were detected as positive. *T. gondii* cyst formation in the brain and reproductive tissues has been reported as more prevalent in sheep and goats than in cattle [31], with the role of beef cattle in the epidemiology of *T. gondii* still unclear. However, it is important to recommend the consumption of cooked beef and avoid feeding the tissues of cattle to pets.

The results from wild animals revealed that only skunks were positive for *T. gondii*, while other scavenger species, such as raccoons or opossums, expected to amplify by PCR, did not. An experimental infection demonstrated that skunks developed fatal toxoplasmosis when fed oocysts, while ingestion of tissue cysts was not fatal [32]. In contrast, another experimental study infecting raccoons with oocysts or tissue cysts resulted in clinically normal animals with antibody development but no detection in tissues [33]. Our results suggest that infected skunks are exposed to oocysts and are more susceptible to developing active infections when compared to other scavenger species. Additionally, skunks had the highest rabies prevalence in this study. This result aligns with what was anticipated since skunks are known to be the main reservoir for the rabies virus in the state of Oklahoma [34]. Other hosts such as dogs, cats, and cattle that tested positive for rabies, had a history of direct contact with skunks. Therefore, we need to emphasize the risk that skunks represent, as well as highlight the importance of rabies vaccinations in domestic animals.

## 5. Conclusions

*T. gondii* and rabies are important zoonoses requiring proper prevention, testing, and accurate diagnosis as part of the One Health approach. In this study, we demonstrated that when ruling out positive rabies cases, *T. gondii* should be tested for due to the similarity in clinical manifestations and tropism for brain tissue. Although no coinfections were found in this study, some limitations should be considered, such as the small tissue section selected for analysis, storage of samples in ethanol solution, and restricted information on anamnesis. Further analysis with different sample storage methods, detailed anamnesis, and expansion in geographic areas would help to understand the prevalence of this parasite in domestic and wild animals when testing for rabies.

## Figures and Tables

**Table 1:** Number of samples tested per host and overall prevalence of *Toxoplasma gondii* and rabies.

Host	Total Tested *n* (%)	*Toxoplasma gondii* Positive *n* (%)	Rabies Positive *n* (%)
Alpaca (*Vicugna pacus*)	2 (0.2)	0	0
Beaver (*Castor canadensis*)	2 (0.2)	0	0
Bovine (*Bos taurus*)	39 (4.3)	2 (0.2)	4 (0.4)
Caprine (*Capra hircus*)	5 (0.5)	0	0
Coyote (*Canis latrans*)	4 (0.4)	0	0
Deer (*Odocoileus virginianus*)	4 (0.4)	0	1 (0.1)
Dogs (*Canis familiaris*)	430 (47.6)	15 (1.7)	5 (0.5)
Equine (*Equus caballus*)	39 (4.3)	0	0
Feline *(Felis catus)*	253 (28.0)	16 (1.8)	3 (0.3)
Gopher (*Geomys bursarius*)	1 (0.1)	0	0
Llama (*Lama glama*)	1 (0.1)	0	0
Mouse (*Mus musculus*)	2 (0.2)	0	0
Opposum (*Didelphis virginianus*)	1 (0.1)	0	0
Ovine (*Ovis aries*)	2 (0.2)	0	0
Porcine (*Sus domesticus*)	2 (0.2)	0	0
Rabbit (*Sylvilagus* spp.)	1 (0.1)	0	0
Raccoon (*Procyon lotor*)	46 (5.1)	0	0
Rat (*Rattus* spp.)	1 (0.1)	0	0
Red fox (*Vulpes vulpes*)	1 (0.1)	0	0
Rhesus macaque (*Macaca mulatta*)	1 (0.1)	0	0
Skunk (*Mephitis mephitis)*	58 (6.4)	3 (0.3)	24 (2.7)
Squirrel (*Sciurus* spp.)	8 (0.9)	0	0
**Total**	903	36	37

**Table 2:** Clinical signs described in hosts testing positive for *Toxoplasma gondii*.

Clinical Sign/ Physical Finding	Bovine (*Bos taurus*)	Dogs (*Canis familiaris*)	Feline *(Felis catus)*	Skunk (*Mephitis mephitis)*
Anorexia	x	x		
Abnormal behavior			x	x
Anemia			x	
Ataxia		x	x	
Aggression	x	x	x	
Diarrhea			x	
Dysphagia			x	
Fever			x	
Hypersalivation	x			
Lethargy			x	x
Nystagmus			x	
Seizures		x	x	
Spasticity			x	
Tremors				x

**Table 3:** Clinical signs described in hosts positive for rabies.

Clinical Sign/ Physical Finding	Bovine (*Bos taurus*)	Dogs (*Canis familiaris*)	Feline *(Felis catus)*	Skunk (*Mephitis mephitis)*	Deer (*Odocoileus virginianus*)
Anorexia	x	x	x		x
Abnormal behavior	x	x	x	x	x
Aggression	x	x	x	x	
Dysphagia		x	x		
Hypersalivation	x	x	x		
Seizures		x	x		
Paralysis		x	x		
Weakness					x

## Data Availability

Patient data is unavailable due to privacy restrictions. All the other data is included in the manuscript.
